# Pharyngeal Colonization by *Kingella kingae,* Transmission, and Pathogenesis of Invasive Infections: A Narrative Review

**DOI:** 10.3390/microorganisms10030637

**Published:** 2022-03-17

**Authors:** Pablo Yagupsky

**Affiliations:** Clinical Microbiology Laboratory, Soroka University Medical Center, Ben-Gurion University of the Negev, Beer-Sheva 84101, Israel; yagupsky@bgu.ac.il

**Keywords:** *Kingella kingae*, colonization, pili, carriage, transmission, children, invasive disease

## Abstract

With the appreciation of *Kingella kingae* as a prime etiology of osteoarticular infections in young children, there is an increasing interest in the pathogenesis of these diseases. The medical literature on *K. kingae*’s colonization and carriage was thoroughly reviewed. *Kingella kingae* colonizes the oropharynx after the second life semester, and its prevalence reaches 10% between the ages of 12 and 24 months, declining thereafter as children reach immunological maturity. *Kingella kingae* colonization is characterized by the periodic substitution of carried organisms by new strains. Whereas some strains frequently colonize asymptomatic children but are rarely isolated from diseased individuals, others are responsible for most invasive infections worldwide, indicating enhanced virulence. The colonized oropharyngeal mucosa is the source of child-to-child transmission, and daycare attendance is associated with a high carriage rate and increased risk of invasive disease. *Kingella kingae* elaborates a potent repeat-in-toxin (RTXA) that lyses epithelial, phagocytic, and synovial cells. This toxin breaches the epithelial barrier, facilitating bloodstream invasion and survival and the colonization of deep body tissues. *Kingella kingae* colonization and carriage play a crucial role in the person-to-person transmission of the bacterium, its dissemination in the community, and the pathogenesis of invasive infections.

## 1. Introduction

Shortly after birth, the skin and the upper respiratory, gastrointestinal, and female genital tracts of a newborn child become gradually coated with a variety of bacterial species, including many potentially dangerous organisms [1]. Despite their distant taxonomic position and vast biological differences, bacteria such as *Haemophilus influenzae* type b and *Streptococcus pneumoniae* colonizing the pharyngeal epithelium display homologous components as pili or antiphagocytic polysaccharide capsules [2]. These striking similarities result from a convergent evolution to adapt to the shared environment, adhere to the mucosal layer, avoid being washed out, and subvert the mucosal immune response.

The composition of the human microbiota is in a continuous dynamic state: organisms are acquired, eradicated, and re-acquired several times in a lifetime, and strains within a given species exhibit a remarkable turnover, indicating that the antigenic variability of virulent factors enables the acquisition of heterologous strains [3,4]. Bacterial colonization is asymptomatic in most cases, and the number of colonized but healthy individuals is enormous compared to those with clinical infections. An invasive disease usually occurs when colonizing bacteria breach the mucosal layer and penetrate the bloodstream. This event may result in the hematogenous dissemination and seeding of the organism to distant sites, causing focal infections. The colonized upper respiratory mucosa is also the source of person-to-person transmission of the bacterium through buccal and respiratory secretions, enabling its spread in the population.

The widespread use of improved culture and molecular detection methods in recent years has resulted in the appreciation of *Kingella kingae* as a common pathogen in young children, causing various invasive diseases involving the skeletal system, bloodstream, and endocardium. This review summarizes the current knowledge of the pharyngeal carriage of this emergent pediatric pathogen, and the dual role of mucosal colonization in the spread of the organism and the pathogenesis of *K. kingae* infections.

## 2. Materials and Methods

The PubMed database was used to search for studies on *K. kingae* colonization and transmission published in the English, Spanish, and French literature between 1 January 1988 and 31 December 2021, using the keywords: “*Moraxella kingii*” or “*Kingella kingae*”, combined with “and” and “colonization”, “carriage”, “transmission”, “infection”, “septic arthritis”, “joint infections“, “bone infections”, “osteomyelitis”, “tenosynovitis”, and “endocarditis’’. Additional papers were also identified from the references lists of articles found in the search, and those of comprehensive review articles on the subject, to make sure that no relevant publications were missed.

## 3. Results and Discussion

### 3.1. Kingella kingae: An Oropharyngeal Resident

Similar to many other members of the *Neisseriaceae* family of Gram-negative organisms, *K. kingae* also colonizes the upper respiratory epithelium. In a large prospective study in which pairs of oropharyngeal and nasopharyngeal specimens were obtained from asymptomatic daycare center attendees, *K. kingae* grew in 109 out of 624 (17.5%) oropharyngeal samples. In contrast, the bacterium was not isolated from the nasopharynx, indicating a restricted ecological niche [5]. This finding was corroborated in a separate study in which 4472 oropharyngeal and nasopharyngeal specimens were sequentially collected from a cohort of 716 young children. Overall, 388 (8.7%) oropharyngeal cultures, but only a single nasopharyngeal culture, recovered *K. kingae* [6].

The colonization of the oropharynx by *K. kingae* organisms plays a double role. On the one hand, the exposed oropharyngeal surfaces are the natural reservoir of the bacterium from which it may be transmitted by contaminated buccal and upper respiratory secretions, and thus disseminated. On the other hand, the colonized oropharyngeal epithelium is the stepping-stone from which the bacterium translocates to the bloodstream and disseminates to remote body sites. In-depth study of the colonization phenomenon is, therefore, crucial to understanding the transmission of *K. kingae* and the pathogenesis of invasive infections.

### 3.2. Mechanism of Colonization

To colonize the oropharynx, *K. kingae* employs type IV pili that anchor the planktonic bacterium to the mucosal surfaces, and thus avoids being removed by saliva and respiratory fluids [7]. Elaboration of these pili is encoded in a chromosomal gene cluster, similar to that found in other Gram-negative pathogens, and in two other genes located in physically separated chromosomal regions, namely *pilC1* and *pilC2* [7,8]. The chromosomal gene cluster consists of a *pilA1* gene that encodes the major pilin subunit and two additional genes, named *pilA2* and *fimB*. The function of *fimB* is unknown, and it does not appear to be required for pilus expression or attachment [7]. The *pilA1* gene sequence shows marked between-strain variation, and the pilA1 subunit exhibits vast differences in antibody reactivity, suggesting that this exposed virulence factor is subjected to selective pressure by the host’s immune system [9]. The pilC1 subunit is needed for twitching motility and adherence, whereas pilC2 has only a minor role in motility and no effect on adherence [8]. The expression of pili in *K. kingae* is finely regulated by the *σ54*, *pilS*, and *pilR* genes [9], and most oropharyngeal isolates and those derived from bacteremic patients express pili. In contrast, those samples isolated from skeletal system infections or endocarditis are usually nonpiliated [10]. This finding suggests that piliation promotes *K. kingae* colonization and facilitates the initial bloodstream invasion but is disadvantageous for invading deep body structures.

In addition to the pili, *K. kingae* elaborates a trimeric autotransporter protein named Knh (*Kingella* NhhA homolog), which is essential for the strong anchoring of the organism to the oropharyngeal epithelium [11]. However, the carbohydrate capsule conceals the Knh element, rendering it inaccessible for attachment to the host’s cells. Porsch et al. have proposed that, following an initial weak adherence of the long pili to the epithelial surface, a strong retraction of these filaments displaces the capsule and exposes the Knh protein, which may then firmly stick to the mucosal surface [11].

### 3.3. Immunity to Colonization and Infection

The crucial role played by the immune system in preventing oropharyngeal *K. kingae* colonization and subsequent invasive disease is supported by the fact that adults with a variety of immunosuppressive conditions are at increased risk of *K. kingae* disease [12]. In a longitudinal study in which serum antibody levels against *K. kingae* outer-membrane proteins were measured by an ELISA test, IgG levels were high at 2 months of age and gradually diminished thereafter, reaching a nadir level at 6–7 months, then remaining low until the age of 18 months, followed by an increase in 24-month-old children. IgA levels were lowest at 2 months and slowly increased between 4 and 7 months of age. A further increment of both antibody types was measured in children aged ≥24 months [13].

This pattern is consistent with protection from colonization and invasive disease by vertically transmitted immunity and limited exposure to *K. kingae* in early infancy. Vanishing maternal antibodies and increasing social contacts result in exposure to the organism in the second life semester, with corresponding increasing IgG and IGA levels. While the colonization and attack rates of disease are high in the second year, antibody levels remain high and stable. Colonization rates, and the incidence of invasive infections and IgG levels decline in older children as they reach immunological maturity. Because asymptomatic *K. kingae* carriage is common in early childhood, whereas invasive infections are exceptional, it is postulated that pharyngeal colonization is the immunizing event.

Similar to other respiratory pathogens such as pneumococci and *H. influenzae* type b, *K. kingae* elaborates a polysaccharide capsule and secretes an exopolysaccharide. Both components inhibit the host’s immune response [14,15,16], enabling colonization of the upper respiratory tract, protecting the organism from phagocytosis by blood leukocytes and tissue macrophages, and facilitating the invasion of deep tissues. The maturation of the T-cell independent arm of the immune system, which is responsible for producing antibodies to polysaccharide antigens, is delayed in humans until the age of 2–4 years [2], explaining the increased susceptibility of young children to both colonization and disease. It should be noted that whereas the prevalence of *K. kingae* colonization reaches its peak and remains steady during the second year of life, the age-related curve of invasive disease is markedly skewed to the left: >75% of affected children are aged <18 months, and >95% are younger than 48 months, indicating that resistance to invasive infections is acquired before immunity to mucosal colonization [12].

### 3.4. Colonization and Transmission

**Figure 1:**
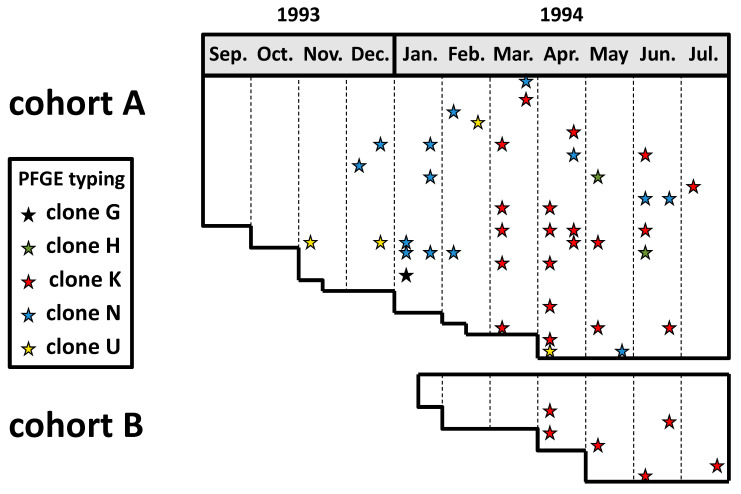
Dissemination of *K. kingae* clones among two cohorts of attendees at an Israeli daycare center. Horizontal lanes: individual attendees. Each star represents a positive pharyngeal culture, while the different colors represent distinct PFGE clones.

A prospective study was conducted in the school year 1993–1994 among two cohorts of young children attending a daycare facility in southern Israel to investigate the dynamics of colonization and transmission of *K. kingae* in children attending out-of-home care facilities [5]. Oropharyngeal specimens were obtained biweekly over 11 months and seeded on the selective blood-agar-vancomycin (BAV) medium [5]. The recovered *K. kingae* isolates were originally studied by pulsed-field gel electrophoresis (PFGE) and ribotyping techniques with multiple restriction enzymes, and immunoblotting with rabbit immune serum [17]. A strict criterion consisting of complete DNA band identity by the three typing methods was employed to characterize the isolates and prove the person-to-person transmission of the strains. More recently, the isolates were retested by PFGE with the highly discriminative *Eag*I enzyme, which is the currently recommended tool for PFGE analysis of the species. The results of this latter analysis are depicted in Figure 1.

Thirty-five of 48 (73%) children carried *K. kingae* organisms at least once, and, on average, 28% of the attendees were colonized at any point in time [5]. Individual attendees exhibited sporadic, intermittent, or continuous carriage, and residing strains were frequently substituted by new strains after weeks or months, showing that similar to other respiratory bacteria, *K. kingae*’s carriage is a dynamic phenomenon with frequent turnover of colonizing organisms [17]. However, it should be pointed out that in the study, a single colony per positive culture was typed, and therefore simultaneous carriage of multiple strains and/or persistence of a previously carried organism at a low and undetectable level cannot be ruled out.

Overall, five distinct PFGE clones were detected in the daycare center during the study period. Four of these clones, namely H, K, N, and U, appeared sequentially in the facility and gradually colonized multiple attendees, while the prevalence of previously carried strains decreased [17] (Figure 1). These observations suggest that prolonged colonization induces an immune response that is strain-specific and eradicates or diminishes the density of the carried strain but does not prevent colonization by an antigenically different organism. Remarkably, although these four highly invasive clones were responsible for over half of the invasive diseases in Israel between 1991 and 2012 [18], none of the colonized daycare attendees developed a clinical *K. kingae* infection during the follow-up period. 

In a secondary analysis performed on the aforementioned southern Israel cohort study [19], the temporal dynamics of *K. kingae* carriage were investigated in children among whom the bacterium was isolated on >1 occasion [6]. The proportion of PFGE-similar strains was determined for pairs of positive cultures separated by ≤2 months (short-term intervals) and for those separated by ≥5 months (long-term intervals). The fraction of pairs of similar strains out of the total number of pairs was assessed by PFGE analysis for short-term and long-term intervals, and then compared. Of the short-term interval paired isolates, 17 of 19 (89.5%) yielded genotypically similar clones, while only 20 of 91 (22.0%) long-term interval pairs yielded genotypically similar clones (*p* < *0.001*), indicating that, over time, colonization enables the eradication of the carried organism, facilitating the later acquisition of a different strain [6].

### 3.5. Detection of K. kingae Colonization

Because of the abundance and complexity of the upper respiratory and buccal microbiota, the isolation and identification of *K. kingae* in oropharyngeal specimens are notoriously tricky. Although the organism frequently colonizes the oropharynx of young children, its presence in Petri dishes is concealed by the rapid overgrowth of other members of the residing bacterial flora [20]. 

**Figure 2:**
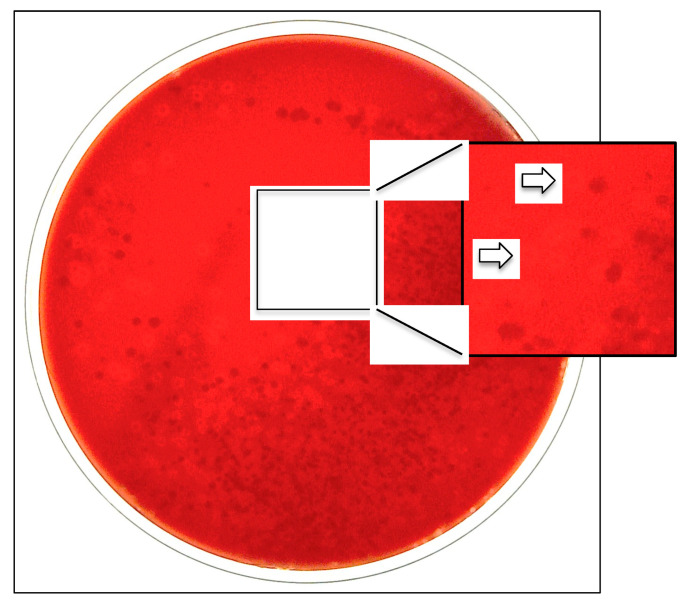
Oropharyngeal specimen seeded onto selective BAV medium exhibiting growth of β-hemolytic *K. kingae* colonies.

To facilitate the culture recovery and identification of the species, a selective and differential medium consisting of blood agar with 2 mcg/mL of added vancomycin (BAV medium) has been developed [20]. BAV plates, streaked with oropharyngeal secretions, are incubated for 48 h at 35 °C under aerobic conditions in a 5% CO_2_-enriched atmosphere [20]. The aerobic conditions suppress the development of anaerobic species; the glycopeptide antimicrobial drug inhibits the growth of Gram-positive bacteria; and the added CO_2_ enhances the growth of capnophilic *K. kingae* organisms, whereas the blood component facilitates the visualization of hemolytic colonies [20] (Figure 2).

When the capability of the BAV and the traditional blood agar media for the primary isolation of *K. kingae* from oropharyngeal cultures were compared in a prospective study, the BAV plate identified 43 of 44 (97.7%) carriers [20]. In contrast, the comparator detected only 10 (22.7%) (*p* < *0.001* by the Chi-squared test) [20]. The BAV medium and a Columbia-agar-based variant [21] have been successfully used in the investigation of the acquisition, prevalence, and transmission of *K. kingae* in the pediatric population and the investigation of outbreaks of invasive disease in daycare facilities [5,6,19,21,22]. It should be pointed out that chocolate agar media are not suitable for *K. kingae* detection in primary cultures since they do not reveal the presence of β-hemolytic colonies. The use of chocolate agar probably contributed to the failure to identify respiratory *K. kingae* carriers in a cluster of infections among attendees at a North Carolina daycare center [23].

In recent years, nucleic acid amplification tests (NAATs) targeting the 16S rRNA or species-specific genes have been introduced into clinical practice to detect fastidious bacteria in normally sterile body fluids and tissues. NAATs enable bacterial identification within hours instead of days and in patients receiving antibiotic therapy [24]. This revolutionary approach is gaining increasing popularity as a sensitive and convenient culture-independent tool for diagnosing invasive *K. kingae* infections [24,25,26,27,28,29,30] and for identifying *K. kingae* carriers in prevalence studies [22,31]. However, because only single genes are amplified, NAATs do not discriminate between different *K. kingae* strains and, thus, have a limited value in investigating complex disease outbreaks [22]. 

Tests that amplify *K. kingae*-specific genes have a higher sensitivity than those targeting the broad spectrum 16S rRNA gene [24]. The three species-specific genes that are targeted by the current assays are the *rtx* operon that encodes the RtxA toxin [32], chaperonin 60 (the *cpn60* gene, also known as *gr*o*EL*) [24], and the malate dehydrogenase (*mdh*) gene [28]. The *rtx*-based tests do not discriminate between *K. kingae* and the recently described *Kingella negevensis* species that also colonizes the pediatric oropharynx [28], and those that amplify the *cpn60* target show suboptimal sensitivity due to variability in the gene sequence among *K. kingae* strains [28]. The novel molecular assay that targets the *mdh* gene exhibits an optimal sensitivity and specificity and will probably replace the older tests [28]. 

### 3.6. Risk Factors for Carriage and Transmission

#### 3.6.1. Age

In a study aimed to identify risk factors for *K. kingae* carriage, multivariate analysis showed that age 6–29 months is strongly and independently associated with oropharyngeal colonization [33], overlapping with the age group with the highest attack rate of invasive infections [34]. The prevalence of pharyngeal carriage among the adult population is low and transient [12]. It usually results from frequent and intimate exposure to young children [35], and the clinical spectrum of invasive *K. kingae* disease is similar to that observed in the pediatric population, affecting especially large joints and the endocardium [12].

**Figure 3:**
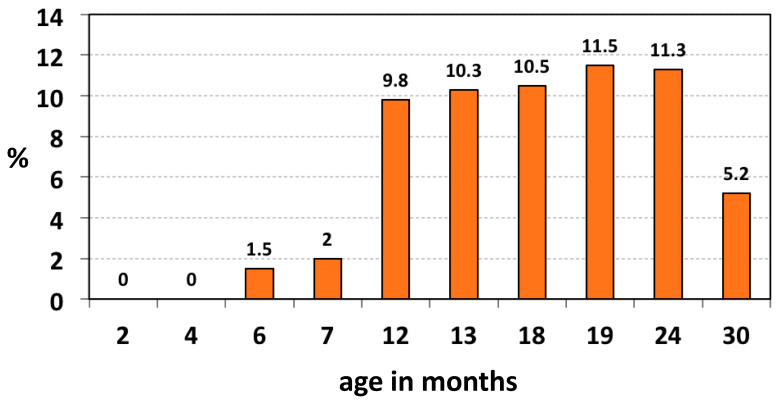
Age-related prevalence of oropharyngeal *K. kingae* colonization.

In a study of the age-related oropharyngeal carriage of *K. kingae* in the healthy children attending a Well-Baby Care Clinic, the organism was not isolated in infants aged < 6 months, and the prevalence increased to 10% in the 6–48 months age group and fell to 6% in older children [5]. In a second study, oropharyngeal specimens submitted to a clinical microbiology laboratory to isolate *Streptococcus pyogenes* were also seeded onto BAV plates [36]. The prevalence of *K. kingae* showed a statistically significant decline with increasing age: it was 3.2% (22 of 694) in children aged <4 years, 1.5% (10 of 679) in the 4–17 years group, and 0.8% (5 of 671) among adults (*p* < *0.001* for trend) [36]. In a longitudinal study aimed to investigate the temporal dynamics of *K. kingae* acquisition and carriage in the young pediatric population, a cohort of 716 Israeli children was sequentially screened between the ages of 2 and 30 months by employing the BAV tool [6]. *Kingella kingae* was not detected below 6 months of age, gradually increased in the 6–12-months interval, reached a zenith between 12 and 24 months of age, and declined significantly at 30 months (*p* < *0.001*) [6] (Figure 3).

#### 3.6.2. Season

Studies conducted in Europe and Israel have consistently shown a significant excess of invasive *K. kingae* infections in the autumn and winter months [36,37]. In a study meant to determine if the incidence of *K. kingae* infections reflects seasonal variation in the colonization rates, oropharyngeal specimens were obtained between February and May, when invasive *K. kingae* morbidity is the lowest, and during the October–December months, representing the annual period with the highest attack rate of clinical infections [36]. Between February and May, 21 of 1020 (2.1%) cultures and 16 of 1024 (1.6%) of those processed in the October–December period were positive for the bacterium (*p* = *0.4*). The lack of seasonality in the colonization rate was also noted in a Swiss study [38], suggesting that the higher attack rate of clinical *K. kingae* infections in the colder months could result from the increased incidence of viral respiratory infections.

#### 3.6.3. Living Conditions, Prevalence, and Transmission

Living standards are known to influence the prevalence and transmission of respiratory organisms and the incidence of associated clinical infections. Southern Israel is populated by Jewish and Bedouin ethnic groups living in dissimilar socioeconomic conditions. On average, the Jewish majority is primarily urban, lives in small family units, and attends daycare centers from an early age [18,19]. The Bedouin minority has recently abandoned its nomadic lifestyle and settled in separate townships where families cluster according to the traditional tribal affiliation. Poverty, low educational levels, and overcrowding are prevalent in the Bedouin communities, and attendance at out-of-home childcare facilities is uncommon. Similar to populations in the developing world, Bedouins experience early acquisition and higher colonization rates by potential respiratory pathogens and increased morbidity and hospitalization rates for infectious diseases [18]. Social mingling between members of the two ethnic groups is limited, and contact between young Jewish and Bedouin children practically does not occur. 

A large cohort study investigated the patterns of *K. kingae* carriage and transmission among healthy children of the two population groups [19]. At 13 months of age, 65 of 316 (20.6%) Bedouin children, but only 46 of 363 (12.7%) Jewish children, had already been colonized by the bacterium at least once (*p* = *0.008*), suggesting that unfavorable living standards facilitate the early acquisition of the organism [19].

**Figure 4:**
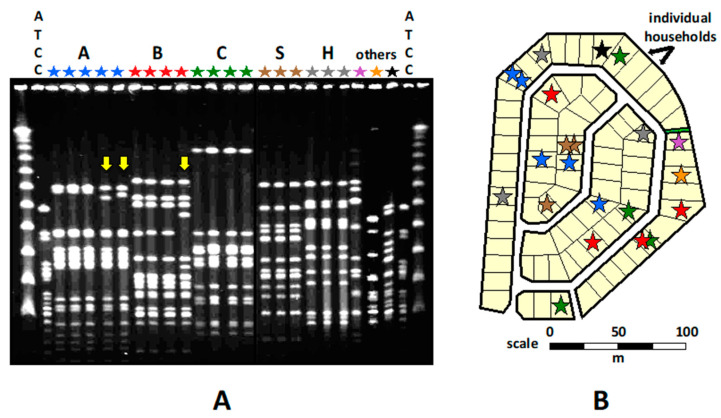
*Kingella kingae* clones carried in a Bedouin town neighborhood, as determined by PFGE with restriction enzyme *Eag*I. (**A**) Capital letters: individual clones; l: size marker; ATCC: ATCC 23,330 *K. kingae* strain; Yellow arrows: clones that exhibit differences in DNA band patterns. (**B**) Spatial distribution of the clones. Each star represents a positive pharyngeal culture, while the different colors represent distinct PFGE clones.

A statistically significant spatial aggregation of *K. kingae* clones was noted in the Bedouin households and neighborhoods, indicating child-to-child transmission through close contact (Figure 4).

Over time, the DNA band patterns of several PFGE clones exhibited subtle changes, indicating gradual accumulation of mutations and/or horizontal gene transfer events in the course of protracted carriage and repeated transmission [19] (Figure 4). The Jewish children also shared similar PFGE clones, but no geographical clustering of PFGE clones was observed. It was suggested that the Westernized Jewish children were connected by numerous and intricate social networks through which *K. kingae* may circulate, obscuring the link between place of residence and spatial distribution of the carried strains [19]. 

Commensal organisms detected in the Bedouin children were generally genotypically different from those isolated from the Jewish children, confirming the lack of social interaction between the two ethnic communities and the importance of close contact for transmission. Five PFGE clones combined (namely A, B, C, D, and E) represented 52% of all *K. kingae* isolates recovered from the Bedouin children, but only 15% of those isolated from the Jewish children (*p* < *0.001*), whereas clones J, K, M, and R represented 50% of all isolates from the Jewish children, but only 2% of isolates from the Bedouin children (*p* < *0.001*) [19]. Many recovered *K. kingae* organisms were genotypically identical to strains isolated in the region over the previous 15 years from healthy carriers and children with bacteremia or osteoarthritis [19]. Clustering a significant fraction of isolates in a few genotypic clones suggests that a limited number of successful strains, characterized by high multiplication rates, transmissibility, and/or resistance to host defenses, persisted over time and disseminated widely. In contrast, less fit organisms failed to propagate or did not circulate for long [19].

#### 3.6.4. Daycare Center Attendance

Over the last decades, a growing proportion of mothers have entered the workforce in industrialized countries, and, as a consequence, a large number of young children attend out-of-home care facilities [39]. This trend has important public health implications because the incidence of infectious diseases, in general, and those caused by respiratory pathogens, particularly, are increased among daycare center attendees [38,39]. In addition to the biological features of the organisms and the immunological immaturity of the young hosts, many additional factors contribute to the enhanced colonization, transmission, and disease rates recorded in childcare facilities, such as the classroom population size and degree of crowding, attendees’ age, the effectiveness of the ventilation system, the length of time spent in the daycare center, or the occurrence of respiratory viral infections [39,40,41,42,43]. Virulent bacteria such as *S. pneumoniae* or the meningococcus may easily spread through contaminated fomites among immunologically naive youngsters, causing disease outbreaks [39,40,41,42].

The link between out-of-home care and *K. kingae* colonization was demonstrated in a prevalence study among 1277 children aged <5 years referred to an Israeli hospital emergency department [33]. Daycare attendance remained strongly and independently associated with *K. kingae* carriage after controlling for other variables (odds ratio 9.66 (95% CI 2.99–31.15), *p* < *0.001*) [33]. Surveillance studies have shown that daycare center attendees frequently harbor genotypically identical organisms and have also demonstrated that carried strains differ between neighboring facilities, indicating that each classroom behaves as a separate and distinct microenvironment [44,45,46]. The role of daycare center attendance in the dissemination of *K. kingae* has been corroborated in a Norwegian study employing NAATs [47]. While 22 of 33 (67%) attendees had detectable *K. kingae* DNA sequences in their oropharynx, only 14 of 165 (9%) children of comparable age not attending out-of-home childcare facilities had a positive test result (*p* < *0.001*) [47].

The importance of close contact between susceptible young children as the driving force for transmission and acquisition of *K. kingae* is backed by the fact that four out of the six outbreaks of invasive disease detected in Israel occurred in daycare centers located in close communities—three military bases and one rural commune (“kibbutz”) [48]. The young population of these hamlets lives within a short distance and spends many hours together, sharing the same classes at daycare facilities, schools and afternoon activities, as well as weekend recreational activities. Thus, each daycare center in this unique epidemiological setting represents a “close community within a close community” that facilitates the circulation of *K. kingae* through prolonged and intimate social mingling [48].

To date, 25 clusters of invasive *K. kingae* disease involving 68 attendees to daycare centers have been reported in the medical literature [49,50,51]. Eleven were detected in France, six in Israel, four in the USA, and two each in Spain and Luxemburg [49,50,51]. In these outbreaks, multiple cases of infection (mean ± SD: 2.7 ± 0.9, range: 2–5 attendees) were simultaneously or sequentially detected over less than a one-month period. Frequently, these events were preceded by outbreaks of viral upper respiratory infections, particularly hand-foot-and-mouth disease and herpangina [49]. Affected children exhibited the whole spectrum of *K. kingae* disease, including septic arthritis, osteomyelitis, spondylodiscitis, tenosynovitis, bacteremia with no focus, endocarditis, and meningitis [49,50,51]. The patients’ average age was 15.1 ± 4.7 months, with a median age of 15 months and a range of 8–32 months, and the mean attack rate in the affected classrooms was 16.9 ± 6.1% (median 15.5%) with a range of 5.6–33.3%. Each outbreak was caused by a single strain, which usually belonged to the internationally distributed sequence type organisms ST-6, STC-14, ST-21–23, or ST-35 [49]. Epidemiological investigation of the outbreaks revealed that the causative strains had widely disseminated in the facility and, on average, 54.2 ± 26.2% of the attendees were colonized (median 55.6%, range 11.8–93.3%) [49,50,51].

Because a carrier’s risk of developing an invasive *K. kingae* infection is low [52], there is no consensus on the need to eradicate the organism from the colonized oropharyngeal mucosa of healthy children attending the affected classrooms. However, in the setting of an explosive outbreak involving multiple attendees to the same daycare facility, prophylactic antibiotics have usually been administered to curtail the event [22,53]. Rifampin was initially chosen because *K. kingae* is uniformly susceptible to the antibiotic [54], and the drug reaches high concentrations in the upper respiratory epithelium and is secreted in saliva [22]. Rifampin administration also had proven efficacy in eradicating meningococcal and *H. influenzae* type b colonization and preventing disease in childcare facilities [39]. However, because, initially, only partial success was achieved with rifampin monotherapy [43], this antibiotic was combined with high-dose amoxicillin in later outbreaks [22,23,52]. Although administration of antimicrobial prophylaxis decreased *K. kingae*’s carriage rate, no complete eradication was achieved, resulting in further dissemination of the invasive strain [43,44,45]. The persistence of the organism in the facilities was not the result of bacterial resistance to the administered antibiotics [23,44,45,53]. Instead, poor compliance and/or failure to administer prophylactic therapy to family contacts could have caused only partial suppression of the reservoir [39]. Notably, despite the incomplete eradication of the invasive organism, no additional disease cases were diagnosed in the affected daycare classrooms [43,44,45]. Extinction of the precipitating viral infection and prolonged *K. kingae* carriage of the invasive strain may have induced herd immunity and prevented new infections [52]. The institution of adequate infection control measures such as improved hygiene and cleaning of shared toys and other objects could also have avoided further pathogen dissemination [52].

#### 3.6.5. Family Transmission

In a Norwegian cross-sectional study, the age-dependent susceptibility to *K. kingae* colonization of the oropharynx was studied by molecular methods [47]. The prevalence of carriage was similar in preschool children without older siblings and in children with siblings. In a second investigation in which Israeli children attending daycare facilities were enrolled, in six of the seven families in which children were discordant for colonization, the carrier was the youngest sibling, confirming that the susceptibility to *K. kingae* colonization diminishes with increasing age [48].

Despite the clear evidence of *K. kingae* transmission between siblings, one may ask why no family outbreaks of disease have ever been reported. It should be realized that children in daycare are segregated by age, and, therefore, classes comprise a large and relatively homogeneous population of attendees with similar degrees of immunological immaturity and susceptibility to infection, whereas in families, at any given time, only a fraction of siblings belong to the high-risk 6–48 months age group.

#### 3.6.6. Carriage in Different Populations

Studies have shown a pediatric carriage rate of 23% in Christchurch, New Zealand [55], 13% in Western Norway [47], 10% in Southern Israel [6,19], 9% in Geneva, Switzerland [52], and 5% in the Paris region [56], but nil in Vancouver, Canada [31] and in Sidney, Australia [57]. Although the wide range of colonization rates found in these studies may indicate actual disparities, factors such as the age of the studied populations, daycare attendance patterns, recent antibiotic exposure, the specimen collection technique, or the sensitivity of the detection method (culture-based or NAATs) may explain some of these discrepancies. While the Israeli and Australian studies were based on pharyngeal cultures, all others employed NAATs. Remarkably, the use NAATs detected *K. kingae* in 11 out of 217 (5.1%) children in the French study, whereas the parallel cultures failed to isolate the organism in all cases [56]. Employing a vancomycin-containing agar plate, Olijve et al. recovered *K. kingae* in only 4 out of 176 (2.3%) New Zealand children aged 6–48 months [55]. In addition, oropharyngeal samples from 48 children between 12 and 24 months of age were used to compare the performance of culture and NAAT for detecting *K. kingae* colonization. While the culture identified two carriers, the molecular method detected nine [55]. The NAAT method had a sensitivity of 82% compared to 18% for cultures, indicating that the culture detection of *K. kingae* colonization is suboptimal [55].

### 3.7. Colonization and Invasive Disease

#### 3.7.1. Carriage Density and Disease

As measured by a quantitative PCR assay, the oropharyngeal colonization density does not vary by age [58], and, contrary to observations made with other respiratory pathogens such as pneumococci and *H. influenzae* type b, it does not differ between diseased individuals and asymptomatic carriers [25]. Thus, while colonization of the oropharyngeal epithelium is an essential precondition for developing the invasive disease, contributory factors other than the bacterial burden, such as upper respiratory viral diseases, probably mediate the transition from asymptomatic colonization to invasive infection.

#### 3.7.2. *Kingella kingae* Colonization: A Stepping Stone of Invasive Infections

Most human diseases caused by members of endogenous microbiota follow a two-stage sequence in which organisms thriving on the different body surfaces translocate to the bloodstream and deep tissues, causing local and/or systemic infections. Although the residing bacterial flora includes many potentially harmful organisms, clinical disease occurs in only a tiny minority of colonized individuals. In a Swiss study in which the prevalence of *K. kingae* in the oropharynx was assessed by a sensitive NAAT, a carriage rate of 9% was found, but bone or joint infections occurred in only 0.8 % of colonized children over a one-year follow-up [52]. This observation is undoubtedly related to the vital protection provided by the host’s immune system, but also to the fact that causing disease may be detrimental to the pathogen. By invading the bloodstream and deep-sited organs, bacteria lose contact with the body surfaces, and, thus, cannot disseminate any further. Sick persons are isolated from healthy individuals, treated with effective antibiotics, and may even die from the infection, disrupting the chain of person-to-person transmission and causing the extinction of the microorganism. Therefore, asymptomatic colonization appears as the optimal relationship for both host and pathogen.

The crucial role of pharyngeal carriage in the pathogenesis of *K. kingae* disease has been convincingly demonstrated in culture- [59] and molecular-based studies [21,60]. Isolates from the oropharyngeal cultures of children with invasive diseases are genomically identical to those recovered from infected body sites, suggesting that the colonized upper respiratory mucosa is the portal through which virulent *K. kingae* strains enter into the bloodstream, disseminate, and invade the skeletal and endocardial tissues, for which the organism shows a particular and still unexplained tropism [21,59,60]. Although *K. kingae* is capable of causing invasive diseases in adults, the overwhelming majority of cases occur in young children, and the age-related epidemic curve of pediatric infections [34] shows a noticeable similarity to that of pharyngeal carriage [6], supporting the concept that infection is an unfortunate consequence of upper respiratory tract colonization.

#### 3.7.3. Viral Infections and *K. kingae* Disease

Invasive *K. kingae* disease is frequently associated with precedent or concomitant symptoms of an upper respiratory infection, such as rhinorrhea, pharyngitis, and cough [12]. In a prospective observational study, Basmaci et al. found viral coinfections in 19 out of 21 (90.5%) children with *K. kingae* osteoarthritis, but in only 3 out of 8 children infected by other bacterial pathogens [61]. Human rhinoviruses were the predominant coinfection, followed by adenoviruses, influenza, parainfluenzae, enteroviruses, and coronavirus OC43 [61]. Buccal erosions caused by primary herpetic gingivostomatitis, hand-foot-and-mouth disease, herpangina, or varicella have also been reported in sporadic cases and outbreaks of *K. kingae* infections [12]. It is postulated that viral diseases play an important role in the pathogenesis of *K. kingae* disease by disrupting the integrity of the oropharyngeal and buccal mucosae, easing the bloodstream invasion by colonizing *K. kingae* organisms. Remarkably, no cases of *K. kingae* disease have been described as a complication of COVID-19 infections.

The importance of viral infections in the dissemination of *K. kingae* among susceptible young children and its role in invasive disease pathogenesis is extensively discussed in an accompanying article in this Special Issue [62].

#### 3.7.4. Strain Diversity

Similar to other members of the *Neisseriaceae* family, *K. kingae* is naturally transformable, and horizontal gene transfer is an essential source of the genomic variability of the species and the emergence of new strains. Currently, >70 sequence types (STs), assembled in 12 ST complexes (STCs) and characterized by distinct allele combinations of 6 housekeeping genes, have been identified by multilocus sequence typing (MLST) [63,64,65]. Employing the PFGE tool, 74 distinct clones, defined by similar DNA bands fingerprints, have also been described, as have 18 *rtxA* and 11 different *por* alleles [63,64]. While some *K. kingae* strains are encountered sporadically and exhibit a restricted regional distribution, others have spread extensively. Moreover, a few identical strains have been recovered from carriers and infected patients in Israel, Western Europe, and North America, where they remained genomically unchanged over long periods, indicating remarkable epidemiologic success and genomic stability [63,64,65].

Notwithstanding the recombination competency of the species, a strong correlation exists between the results of the various typing schemes, even though these methods analyze different segments of the bacterial genome. For instance, all PFGE clone K organisms studied to date belong to the multilocus strain typing (MLST) STC-6 and possess the closely related *rtxA*-8 or *rtxA*-9 alleles and the *por*-1 allele [63,64]. The striking genomic homogeneity found among *K. kingae* clones, disregarding the geographic and/or temporal origins, defies the disrupting effect of lateral gene transfer. It is hypothesized that epidemiologically successful *K. kingae* strains are positively selected because they possess an advantageous combination of genetic traits, maintaining the allelic linkage disequilibrium and ensuring their global dissemination and temporal persistence.

#### 3.7.5. Virulence of Carried Strains

In the carriage study mentioned above conducted in southern Israel, 40 different *K. kingae* clones were identified by PFGE typing among 188 commensal isolates, of which 24 (60%) clones were carried by >1 child and 16 were unique [19]. Whereas some PFGE clones were frequently isolated from the pharynx of asymptomatic children, but rarely detected in infected patients, others were overrepresented among invasive strains, suggesting substantial differences in terms of virulence. The clones A, C, J, and R, common among healthy carriers [19], have seldom been detected in the blood or skeletal system exudates of sick children [65], suggesting a compromise between transmissibility and virulence. These results could also indicate that carriage of the less virulent strains induces a weaker immune response, leading to a more prolonged carriage and overrepresentation in the sampling. In contrast, strains associated with deep-tissue invasiveness may be rapidly eradicated because survival in different body ecosystems requires a specific biological specialization.

In a study in which the clonal distribution of 102 invasive Israeli strains isolated from children with bacteremia, endocarditis, or skeletal system infections were analyzed by PFGE, 19 distinct clones were detected. Five of these clones (namely B, H, K, N, and W) collectively comprised 77% of all isolates, indicating enhanced virulence [65]. Clone K appeared to possess an optimal balance between transmissibility and virulence. Isolates belonging to this clone were commonly carried in the young pediatric population of southern Israel, predominant among attendees at a daycare center in the early 1990s, persisting in the oropharynx for up to four months [17], and were the second most commonly carried clone in a prevalence study conducted in 2006–2007 [19]. On the other hand, clone K organisms were cultured from 42% of all patients with invasive infections in the region over a two-decade period [18,65], and were also a common etiology of disease outbreaks in daycare facilities [53]. Employing an animal model of invasive infection, Basmaci et al. have demonstrated that *K. kingae* organisms exhibit a wide strain-to-strain variation in terms of animal survival, ranging from no apparent disease to a septicemic and rapidly lethal illness [64], confirming the results of studies of carriage and disease in children.

In addition to remarkable invasiveness, a few invasive clones demonstrated tissue tropism at the population level. Clone K showed a statistically significant association with bacteremia with no focal infection, clone N with septic arthritis and osteomyelitis, and clone P with endocardial infections [65]. These results have been reproduced in a large international study in which 324 isolates were typed by MLST and correlated with the patient’s clinical syndrome [63]. ST-35, ST-14, and ST-25 (which correspond to PFGE clones N, H, and Sp, respectively) were significantly associated with skeletal system disease, and ST-24 (PFGE clone P) was associated with endocarditis [63]. These observations imply that the carriage of some *K. kingae* strains entails an increased risk for developing specific clinical diseases.

Epidemiological research conducted in the Negev region of southern Israel has also provided a better understanding of the link between the differences in virulence of *K. kingae* strains and the resulting morbidity rates. Despite the unfavorable socioeconomic conditions of the Bedouin children, the incidence of invasive *K. kingae* infections during a 23 year follow-up was significantly lower than that found among the Jewish children ((5.8/100,000 and 12.2/100,000, respectively) (95% confidence interval of the difference: 1.07–3.60, *p* < *0.05*)) [18]. Isolates derived from the sick Jewish children showed noticeable clustering, and clone K comprised 30 of the 72 (42%) isolates. The distribution of *K. kingae* clones was more scattered in the Bedouin children, no single clone represented >20% of isolates, and clone K was identified in only three isolates (10%) [18]. This differential clonal distribution of invasive *K. kingae* strains between the two ethnic groups was similar to that found among healthy Negev carriers [19]. Clone K organisms were detected in 16 of the 91 (18%) colonized Jewish children, but in only 2 of 149 (1%) Bedouin children (*p* < *0.001*) [19]. These results suggest that the enhanced circulation of the highly virulent clone K among the Jewish population of southern Israel contributes to the increased morbidity observed in this ethnic group [18].

#### 3.7.6. Oropharyngeal Colonization and the Diagnosis of Invasive *K. kingae* Disease

*Kingella kingae* infections of the skeletal system frequently involve small joints and bones that are difficult to access and aspirate [12]. Therefore, specimens from these infected sites are not always available to establish a microbiological diagnosis [21,60]. The problem is particularly troublesome in young children with spondylodiscitis because biopsies or needle sampling of the pediatric spine require general anesthesia, and the yield of these procedures and blood cultures is low [29].

In recent years, an alternate non-invasive diagnostic strategy, consisting of obtaining an oropharyngeal specimen and subjecting it to a sensitive *K. kingae*-specific NAAT, has been recommended [21,29,60]. The patient’s age of <48 months and a benign clinical presentation, combined with a positive NAAT result, implicate *K. kingae* as the probable etiology of the infection [29,60]. Naturally, *K. kingae* detection in the oropharynx of a child with a skeletal system infection is not irrefutable proof of the etiology of the disease because the background carriage rate of the organism in the relevant age group averages 10–12%, and >20% among children attending daycare facilities [12]. On the other hand, because oropharyngeal colonization is a sine qua non for the development of *K. kingae* osteoarthritis, the predictive value of a negative NAAT is high, and failure to detect *K. kingae*’s DNA targets virtually excludes the bacterium as the agent of the disease.

## 4. Conclusions

Intensive research conducted over the last three decades has resulted in recognition of *K. kingae* as a significant invasive pathogen of early childhood, causing bacteremia, septic arthritis, osteomyelitis, and endocarditis in the 6–48 months age group. The bacterium is notoriously difficult to recover in culture, and the use of NAATs has significantly improved its detection in upper respiratory tract specimens and skeletal system exudates. *Kingella kingae* is carried on the oropharyngeal epithelium, and the epidemiological curve of mucosal colonization overlaps with that of clinical infections. The oropharynx is the source of droplet transmission of *K. kingae* among susceptible young children and its dissemination in the population. The colonized mucosal surface also plays a crucial role in the pathogenesis of clinical infections, being the portal of entry to the bloodstream from which it spreads through the hematogenous route, invading joints, bones, and the endocardial tissues for which the organism exhibits a particular tropism.
